# Drug landscape in patients receiving general outpatient palliative care in Germany: results from a retrospective analysis of 10,464 patients

**DOI:** 10.1186/s12904-023-01231-3

**Published:** 2023-08-19

**Authors:** Sven H. Loosen, Jacqueline Schwartz, Steven Grewe, Sarah Krieg, Andreas Krieg, Tom Luedde, Yann-Nicolas Batzler, Karel Kostev, Martin Neukirchen, Christoph Roderburg

**Affiliations:** 1 Department of Gastroenterology, Hepatology and Infectious Diseases, Medical Faculty of Heinrich, University Hospital Duesseldorf, Heine University Duesseldorf, Moorenstraße 5, 40225 Duesseldorf, Germany; 2 Interdisciplinary Center for Palliative Medicine, Medical Faculty of Heinrich, University Hospital Duesseldorf, Heine University Duesseldorf, Moorenstrasse 5, 40225 Duesseldorf, Duesseldorf, Germany; 3 Department of Surgery (A), Medical Faculty, University Hospital Duesseldorf, Heinrich-Heine- University Duesseldorf, Moorenstrasse 5, 40225 Duesseldorf, Germany; 4 Epidemiology, IQVIA, Frankfurt, Germany; 5 Department of Anesthesiology, Medical Faculty of Heinrich, University Hospital Duesseldorf, Heine University Duesseldorf, Moorenstrasse 5, 40225 Duesseldorf, Germany

**Keywords:** Outpatient palliative care, Prescription, Medication, Deprescription, Opioids, Sedatives, Antiemetics

## Abstract

**Background:**

According to § 27 and § 87 1b of the German Social Code, Book V, general outpatient palliative care (GOPC) aims to promote, maintain, and improve the quality of life and self-determination of seriously ill people. It should enable them to live in dignity until death in their preferred environment. Instead of a curative approach GOPC treatment focuses on the multiprofessional objective of alleviating symptoms and suffering on a case-by-case basis using medication or other measures, as well as the management of an individual treatment plan. The aim of this study was therefore to investigate to what extent medication differs from 12 months prior GOPC treatment within 12 months following GOPC treatment.

**Methods:**

A retrospective database cross sectional study based on the IQVIA Disease Analyzer (DA) was performed, including adult patients with cancer diagnosis and at least one documentation of palliative support between January 1st, 2018 and December 31st, 2021, in 805 general practices (GP).

**Results:**

The results of this study show, that in the context of general general outpatient palliative care, there is a significant increase in the prescription of opioids (18.3% vs. 37.7%), sedatives (7.8% vs. 16.2%) and antiemetics (5.3% vs. 9.7%), as well as a significant reduction in other medications such as statins (21.4% vs. 11.5%), proton pump inhibitors (PPI) (41.2% vs. 35.3%), or antihypertensives (57.5% vs. 46.6%).

**Conclusions:**

Our results support the role of GOPC as an important element in improving pharmacological symptom control and deprescription to improve quality of life of patients at the end of their life.

**Supplementary Information:**

The online version contains supplementary material available at 10.1186/s12904-023-01231-3.

## Background

Today, palliative care aims to alleviate the consequences of an illness when there is no longer any prospect of a cure [1, 2]. While until recently palliative care was classically practiced as a medical discipline in hospitals or hospices, thus in the inpatient setting, recent years have seen an expansion of palliative care into the outpatient setting. This obviously poses major challenges to existing primary care structures, which are not designed for the complex, multidisciplinary, and very time-intensive care of palliative patients [3–5]. In order to cope with these challenges, the German Social Code Book V (Sozialgesetzbuch V), provides for general outpatient palliative care (GOPC), with the aim of maintaining, promoting and improving the quality of life and self-determination of palliative patients as far as possible enabling them to live in dignity until death in their familiar surrounding [6]. Reflecting the complex clinical, psychosocial and spiritual situation of patients at the end of their life, within the GOPC system, patients are cared for by specially trained caregivers including family practitioners with a focus on palliative care medicine [7–9]. By 2021, 14,620 physicians had completed additional training in palliative medicine [10] enabling them e.g. to work within a specialized outpatient palliative care team. On the patient side, tumor diseases continue to be the most common reason for palliative care [11].

Since many years large resources have gone into establishing and operating general and specialized GOPC structures in Germany. In contrast, there are only few evaluations of this system. In particular, data are lacking on whether the involvement of GOPC resources lead to a concrete change in care of patients, as expressed, for example, by an adaptation of medication to the specific palliative care situation. The aim of this study was to investigate to what extent medication differs from 12 months prior GOPC treatment within 12 months following GOPC treatment.

## Methods

### Data source

This study represents a retrospective database cross sectional study based on the IQVIA Disease Analyzer (DA) database, which contains case-based information including demographic data, medical diagnoses, and prescription information provided by office-based physicians (general practitioners and specialists) in Germany. The quality of the data is regularly assessed by IQVIA on a number of criteria (e.g., completeness of documentation and linkage between diagnoses and prescriptions). It has been previously found that the panel of practices included in the DA database is representative for the general and specialized practices in Germany [12].

### Study population

This study included adult individuals (18 years or older) in 805 general practices (GP) with at least one documentation of palliative support between January 1st, 2018 and December 31st, 2021 (index date) as well as a cancer diagnosis (ICD-10: C00-C97) 30 days prior to or at the index date. GOPC support was considered using billing numbers according to the appropriate value measurement (German: EBM) and the fee regulations for doctors (German: GOÄ) including 03370, 03371, 03372, 03373 (Supplementary Table 1).

### Study outcomes

The first outcome of this study were proportions of different therapies prescribed by GPs among patients receiving palliative outpatient care within 12 months prior to the index date and within 12 months following the index date. Differences between medication proportion prior versus after the index date were assessed using McNemar tests. P-values < 0.05 were considered statistically significant. Additionally, treatments prescribed after the index date were shown for all patients in total as well as five age groups (18–50, 51–60, 61–70, 71–80, > 80 years), women and men, and the most frequent cancer diagnoses (digestive organs, respiratory organs, female breast, prostate, and lymphoid and hematopoietic tissue) separately. Treatments analyzed included: opioids (EphMRA ATC: N06A), non-steroid antirheumatics (NSAR) (ATC: M01A/N02B), systemic corticosteroids (ATC: H02), antidepressants (ATC: N06A), antipsychotics (ATC: N05A), hypnotics/sedatives (ATC: N06C), antiepileptics (ATC: N03), proton pump inhibitors (ATC: A02B2), antiemetics and antinauseants (ATC: A04A), drugs for constipation (ATC: A06A), propulsives (ATC: A03F), antihypertensives (ATC: N03, N07, N08, N09), statins (ATC: A10A), thyroid preparations (ATC: H03). Differences between age groups and cancer types were assessed using Chi^2^ tests. P-values < 0.05 were considered statistically significant. Analyses were performed using SAS version 9.4 (Cary, NC: SAS Institute Inc).

## Results

### Patient characteristics

A total of 10,464 cancer patients receiving GOPC in Germany were identified from the Disease Analyzer database within the study period. The mean age (standard deviation (SD)) was 73.2 years (12.6 years). 50.0% of patients were female. Digestive organs cancer was the most prevalent type of cancer (27.2%), followed by respiratory organ (17.6%), breast (13.5%), lymphoid and hematopoietic tissue (11.1%), and prostate cancer (7.4%) (Table 1).

**Table 1:** Baseline characteristics of study patients

Patient group	N (%)
Total	10,464
Age (mean, SD)	73.2 (12.6)
**Age group**	
Age 18–50	480 (4.6)
Age 51–60	1319 (12.6)
Age 61–70	2216 (21.2)
Age 71–80	3010 (28.8)
Age > 80	3439 (32.9)
**Sex**	
Women	5229 (50.0)
Men	5235 (50.0)
**Cancer site**	
Digestive organ cancer	2848 (27.2)
Respiratory organ cancer	1846 (17.6)
Breast cancer	1412 (13.5)
Prostate cancer	774 (7.4)
Lymphoid and hematopoietic tissue cancer	1161 (11.1)
All other cancers	2423 (23.1)

### Therapies prescribed during outpatient palliative care

**Fig. 1:**
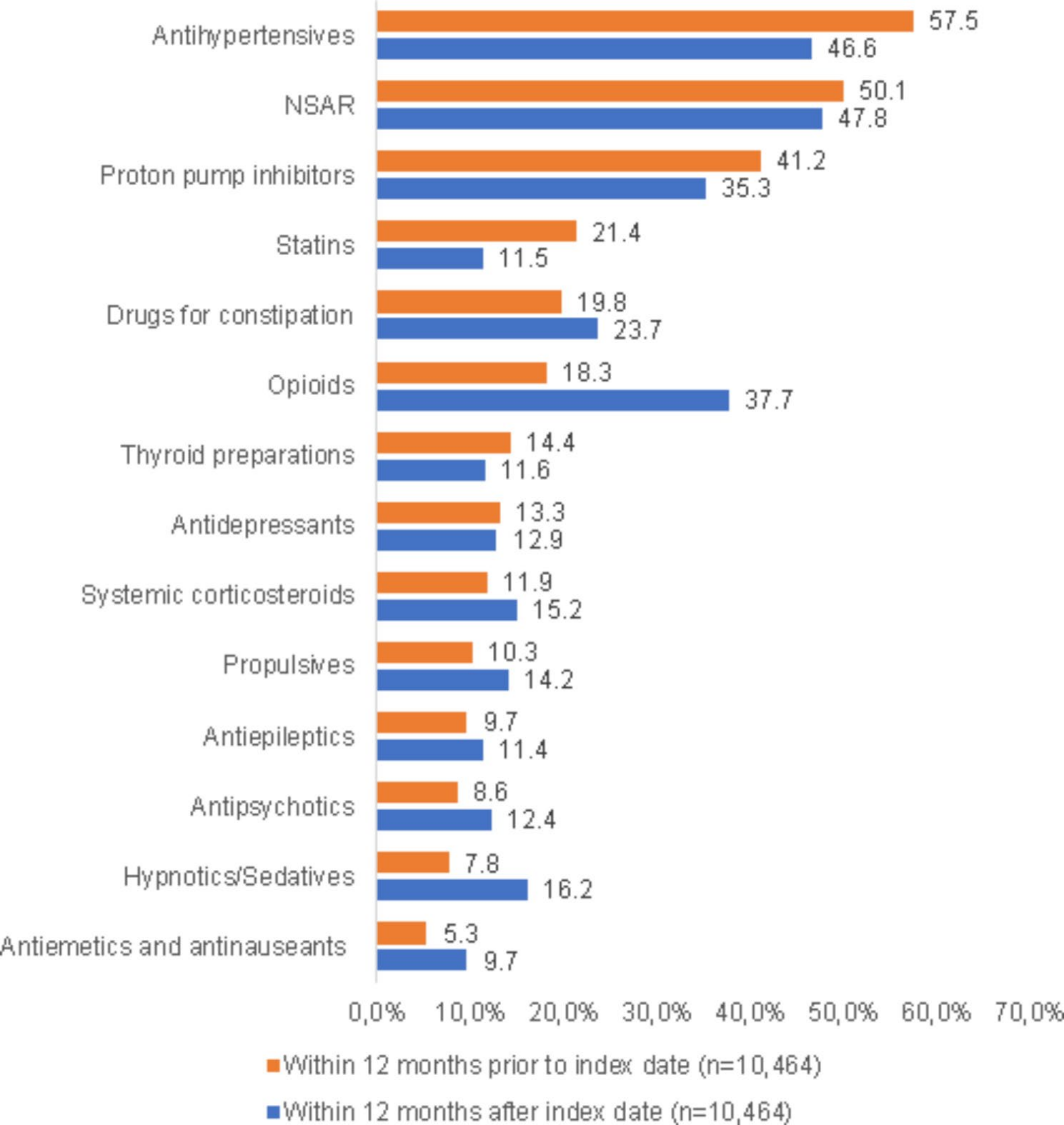
Therapies prescribed by GPs among patients receiving general palliative outpatient care within 12 months prior and within 12 month after first palliative care notice

When comparing prescriptions before and after the initiation of palliative outpatient care, we observed that the proportions of patients who received opioids (18.3% vs. 37.7%), drugs for constipation (19.8% vs. 23.7%), hypnotics/sedatives (7.8% vs. 16.2%), systemic corticosteroids (11.9% vs. 15.2%), propulsives (10.3% vs. 14.2%), antipsychotics (8.6% vs. 12.4%), antiepileptics (9.7% vs. 11.4%) and antiemetics/antinauseants (5.3% vs. 9.7%) increased within the first year (Fig. 1). In contrast, the proportions of patients receiving antihypertensives (57.5% vs. 46.6%), NSARs (50.1% vs. 47.8%), proton pump inhibitors (41.2% vs. 35.3%) or statins (21.4% vs. 11.5%) decreased following the initiation of palliative care (Fig. 1).

### Age-related differences of palliative therapy prescriptions

Although there were several significant differences between age groups in terms of prescribed therapies, clear positive age-related trends were observed for antipsychotics (from 7.1% in the age group 18–50 years to 15.8% in the age group > 80 years), constipation drugs (from 17.5 to 25.2%) as well as antihypertensives (from 22.5 to 52.3%, Table 2). Contrarily, we observed a negative age-related trend for antiemetics and antinauseants (from 13.5% in the age group 18–50 years to 7.2% in the age group > 80 years) as well as antiepileptics (from 15.2 to 8.0%, Table 2).

**Table 2:** Proportion of different therapies prescribed by GPs among patients receiving general palliative outpatient care within 12 month after first palliative care notice by age group

Therapy	Age 18–50	Age 51–60	Age 61–70	Age 71–80	Age > 80	Chi ^2^ test (p-value)
Opioids	39.8	35.3	37.6	38.4	39.1	< 0.001
Hypnotics/Sedatives	13.8	15.5	16.8	15.8	16.8	0.364
Antipsychotics	7.1	10.1	10.0	12.1	15.8	< 0.001
Propulsives	13.3	15.5	15.8	14.3	12.7	0.010
Systemic corticosteroids	15.8	19.4	19.1	15.4	10.8	< 0.001
Drugs for constipation	17.5	21.5	23.7	24.1	25.2	0.001
Antiemetics and antinauseants	13.5	12.8	11.9	9.2	7.2	< 0.001
Antiepileptics	15.2	15.9	13.7	11.0	8.0	< 0.001
Antihypertensives	22.5	34.6	45.0	50.4	52.3	< 0.001
Proton pump inhibitors	34.0	37.7	38.9	36.3	31.5	< 0.001
Antidepressants	14.4	14.6	13.3	13.0	11.9	0.104
Thyroid preparations	12.1	11.5	11.8	13.2	10.1	0.003
Statins	2.3	7.5	11.4	13.7	12.5	< 0.001
NSAR	46.1	47.2	49.4	47.1	47.8	0.483

### Sex-related differences of palliative therapy prescriptions

The prevalence of most treatments during palliative care were comparable between women and men. Significant differences were observed for propulsives (16.1% in women; 12.4% in men), antiemetics and antinauseants (11.6% in women; 8.0% in men), antidepressants (15.0% in women; 10.8% in men), thyroid preparations (15.8 in women; 7.4% in men), as well as statins (8.8% in women; 14.3% in men, Table 3).

**Table 3:** Proportion of different therapies prescribed by GPs among patients general receiving palliative outpatient care within 12 month after first palliative care notice by gender

Therapy	Women	Men	Chi ^2^ test (P value)
Opioids	37.9	37.5	0.728
Hypnotics/Sedatives	17.0	15.3	0.017
Antipsychotics	12.7	12.0	0.276
Propulsives	16.1	12.4	< 0.001
Systemic corticosteroids	15.1	15.3	0.721
Drugs for constipation	24.0	23.4	0.456
Antiemetics and antinauseants	11.6	8.0	< 0.001
Antiepileptics	11.4	11.4	0.992
Antihypertensives	46.5	46.7	0.888
Proton pump inhibitors	35.8	34.8	0.325
Antidepressants	15.0	10.8	< 0.001
Thyroid preparations	15.8	7.4	< 0.001
Statins	8.8	14.3	< 0.001
NSAR	48.2	47.4	0.447

### Cancer site-related differences of palliative therapy prescriptions

Table 4 shows the proportions of prescribed therapies stratified by cancer site. Opioids (42.6%), systemic corticosteroids (23.3%), drugs for constipation (26.7%), and hypnotics/sedatives (18.9%) were prescribed more often in patients with respiratory organ cancer compared to the other cancer sites (Table 4). Propulsives (18.4%) and proton pump inhibitors (38.5%) had a higher prevalence among patients with digestive organ cancer (Table 4).

**Table 4:** Proportion of different therapies prescribed by GPs among patients receiving general palliative outpatient care within 12 month after first palliative care notice by cancer type

Therapy	Digestive organs	Respiratory organs	Female breat	Prostate	Lymphoid and hematopoietic tissue	Chi ^2^ test (P value)
Opioids	37.8	42.6	35.3	40.7	36.8	< 0.001
Hypnotics/Sedatives	16.2	18.9	14.3	15.4	15.0	0.005
Antipsychotics	11.9	11.3	12.7	12.9	14.0	0.233
Propulsives	18.4	15.5	14.6	10.0	11.8	< 0.001
Systemic corticosteroids	10.5	23.3	12.6	14.9	13.8	< 0.001
Drugs for constipation	24.0	26.7	23.7	25.5	20.1	0.001
Antiemetics and antinauseants	11.4	11.7	9.7	7.6	6.3	< 0.001
Antiepileptics	8.2	14.5	11.0	9.7	8.8	< 0.001
Antihypertensives	47.5	46.4	49.2	50.9	44.2	0.025
Proton pump inhibitors	38.5	37.6	36.3	31.8	32.2	< 0.001
Antidepressants	12.2	15.0	15.4	11.0	13.1	0.002
Thyroid preparations	10.0	10.4	14.8	7.6	11.4	< 0.001
Statins	10.5	12.6	10.3	16.9	10.3	< 0.001
NSAR	48.7	50.7	49.1	51.0	42.2	< 0.001

## Discussion

In this study, we demonstrate that the involvement of general outpatient care structures is associated with significant changes in patients’ medications. High medication burden, complex regimens and frequent changes - either an addition of symptom reducing medication or discontinuation of others - are common [13, 14]. Among others, main referral criteria for outpatient palliative care are physical symptoms [11]. Leading symptoms for including palliative care are pain, fatigue, depression, anxiety, sleep [14] and dyspnea according to the underlying diagnosis. To address these uncontrolled symptoms, the patient`s medication profile needs to be adapted. Therefore, an overall increase in medication providing symptom relief could be assumed and is in fact shown in our data, as well as in literature [13]. At the same time a decrease in preventive medication, such as statins, can be observed, as they do not pursue any therapeutic goal of symptom relief. Other medication like antihypertensives can be reduced or discontinued completely, according to an overall deterioration towards the end of life. As an example, concomitant dysphagia, observed in gastrointestinal malignancies or in end of life situations, prevents further oral administration.

With regard to the main physical symptoms, pain is the most notable problem. To reduce pain, especially in palliative care patients, the prescription of opioids is required and frequently established. Hence, data suggest an insufficient prescription prior to GOPC, as a relevant change in pain management is observed in 67.7% [15]. Our findings show an increase in opioids, which has been observed before [16–18]. In the same time NSAR are not likely to contribute to a further symptom improvement, so we can show a moderate decrease in prescription. Opioids are also used in therapy of dyspnea, this indication may come to account, as cancer of respiratory organs or pulmonary metastasis are frequent. Probably due to the opioid induced constipation, an increase in laxatives can be documented. The increase is moderate compared to the opioid increase. In another population this fact was related to an overall low severity constipation that did not require adjustment in medication [13].

The increasing use of systemic corticosteroids has been reported before [13, 19]. Common but unspecified indications are decreased appetite, fatigue, poor wellbeing, nausea and pain management or dyspnea. Our data confirm this finding, even though we cannot point out reasons for the individual prescriptions. Concerning psychopharmacological medication, we detect an increase in prescription of sedatives, antipsychotics and antiepileptics. Antiepileptics are e.g. indicated in treating seizures due to cerebral metastasis, who are likely to occur in lung cancer, breast cancer or less frequently in gastrointestinal tumors. Apart from that, certain antiepileptics may be used additionally in pain management or e.g. in nausea as an off-label-treatment. Antipsychotics are needed for the treatment of a delirium originating from of cerebral metastases or towards the end of life, which explains the documented increase in prescription. As palliative care is a holistic approach to patients’ symptom burden, we find a notably higher rate of prescribed sedatives. Sedatives are used to treat anxiety, either in an earlier stage of illness or to prevent patients from suffering fear or other not manageable symptoms towards the dying process. With regard to the increase of propulsives, they are either used in treating constipation, or in ileus treatment. The latter occurs in gastrointestinal malignancies, who are a frequent diagnosis in our data, or in peritoneal carcinomatosis depending on the underlying tumor.

### Limitations and strengths

Our data underscore the role of GOPC in patients no longer amendable to curative treatment strategies and should form the basis for prospective studies in this area to further improve treatment of chronically ill patients by involving palliative care. It is important to note that our study was limited by some aspects, which are mainly related to the database used and study methods. In brief, all diagnoses are coded using ICD-10 codes, which potentially leads to a misclassification and undercoding of certain diagnoses. Moreover, data on concomitant diseases, the socioeconomic status (e.g., education and income of patients) as well as lifestyle-related risk factors (e.g., smoking, alcohol consumption) are also lacking but might influence the medication of the individual patient. Further on, we are unable to determine the indication for which a certain drug was used in the individual situation. The same remains true for information on the individual patients´ symptom burden (e.g., dyspnea, anxiety, delirium) and stage of illness that would have allowed more detailed analyses. In addition, lab values are documented only in a part of patients potentially introducing another bias. However, the IQVIA Disease Analyzer database that was used for the analyses of this study has proven its statistical validity in numerous previous publications [20–22].

## Conclusion

In conclusion our data highlight the need for an improved symptom control in a large number of outpatient palliative cancer patients. This finding leads to the question, whether palliative patients without GOPC may benefit from improved pharmacological symptom control and deprescription to improve quality of life. Here further studies as well as ongoing medical education are needed to reduce symptom burden earlier. Finally, further studies could further explore patients experiences and preferences regarding medications during pallative care by building upon the findings of this study.

### Electronic supplementary material

Below is the link to the electronic supplementary material.

Supplementary Material 1

## Data Availability

The datasets used and/or analysed during the current study are available from the corresponding author and on reasonable request.

## List of Abbreviations

ATC: Anatomical therapeutic chemical classification system
DA: Disease analyzer
GP: General practice
ICD: International classification system
NSAR: Non steroid antirheumatics
GOPC: General outpatient palliative care
PPI: Proton pump inhibitors
SAS: Statistical analysis system
SD: Standard deviation
