# Supplementary material for: Drug landscape in patients receiving general outpatient palliative care in Germany: results from a retrospective analysis of 10,464 patients
Source: BMC Palliat Care. 2023 Aug 19;22:118. doi: 10.1186/s12904-023-01231-3 (PMC10439623; doi:10.1186/s12904-023-01231-3)
Supplement: Supplementary file 1 — Supplementary Material 1 [file 12904_2023_1231_MOESM1_ESM.docx]

| **Billing number** | **Palliative care treatment** |
| --- | --- |
| 03370 | Initial palliative care assessment of the patient's status incl. treatment plan |
| 03371 | Palliative care treatment in practice in addition to number 0300 (standard remuneration for every insured person) |
| 03372 | Palliative care treatment at home in addition to number 01410 (standard home visit) and 01413 (standard home visit of another patient in the same household) |
| 03373 | Palliative care treatment at home or in a nursing home in addition to number 01411, 01412 or 01415 (urgent home or nursing home visits) |

Suppl. Table 1: Billing number and associated palliative care treatment
